# Multidetector computed tomography angiography for assessment of in-stent restenosis: meta-analysis of diagnostic performance

**DOI:** 10.1186/1471-2342-8-14

**Published:** 2008-07-31

**Authors:** Piet K Vanhoenacker, Isabel Decramer, Olivier Bladt, Giovanna Sarno, Erik Van Hul, William Wijns, Ben A Dwamena

**Affiliations:** 1 Department of Radiology and Imaging, OLV Ziekenhuis, Aalst, Belgium; 2 Cardiovascular Center Aalst, Aalst, Belgium; 3 Division of Nuclear Medicine, Department of Radiology, University of Michigan Health System, Ann Arbor, USA

## Abstract

**Background:**

Multi-detector computed tomography angiography (MDCTA)of the coronary arteries after stenting has been evaluated in multiple studies.

The purpose of this study was to perform a structured review and meta-analysis of the diagnostic performance of MDCTA for the detection of in-stent restenosis in the coronary arteries.

**Methods:**

A Pubmed and manual search of the literature on in-stent restenosis (ISR) detected on MDCTA compared with conventional coronary angiography (CA) was performed. Bivariate summary receiver operating curve (SROC) analysis, with calculation of summary estimates was done on a stent and patient basis. In addition, the influence of study characteristics on diagnostic performance and number of non-assessable segments (NAP) was investigated with logistic meta-regression.

**Results:**

Fourteen studies were included. On a stent basis, Pooled sensitivity and specificity were 0.82(0.72–0.89) and 0.91 (0.83–0.96). Pooled negative likelihood ratio and positive likelihood ratio were 0.20 (0.13–0.32) and 9.34 (4.68–18.62) respectively. The exclusion of non-assessable stents and the strut thickness of the stents had an influence on the diagnostic performance. The proportion of non-assessable stents was influenced by the number of detectors, stent diameter, strut thickness and the use of an edge-enhancing kernel.

**Conclusion:**

The sensitivity of MDTCA for the detection of in-stent stenosis is insufficient to use this test to select patients for further invasive testing as with this strategy around 20% of the patients with in-stent stenosis would be missed. Further improvement of scanner technology is needed before it can be recommended as a triage instrument in practice. In addition, the number of non-assessable stents is also high.

## Background

Coronary artery disease (CAD) remains one of the leading causes of death and morbidity in the Western world. Each year one million patients are treated percutaneously with stent implantation [1]. Unfortunately angioplasty and stent implantation is not a permanently curative treatment and even with drug eluting stents, a substantial fraction of patients will develop recurrent symptoms due to in-stent restenosis (ISR). Cumulative frequency of stenosis immediately after stenting and at six months in patients who received sirolimus-eluting stents and standard stents showed the following pattern: at six months, restenosis, defined as luminal narrowing of 50 percent or more occurred in no patient with a sirolimus stent compared to 23 percent of those with standard stents. The percentage of stenosis at six months with sirolimus stents was essentially the same as that immediately after the procedure and in all cases was less than 35 percent [2]. In a recent meta-analysis, restenosis was also highly reduced from 31.7% with bare stents to 10.5% with DES [3].

Invasive coronary angiography (CA) is the standard of reference for the evaluation of stent patency and the exclusion of in-stent restenosis (ISR). Although it has a small risk of complications it is an invasive procedure with significant costs [4-6]. A non-invasive technique for the assessment of stent patency would therefore be highly desirable.

Multi-detector computed tomography angiography (MDCTA) of the coronary tree has been evaluated in multiple studies, to assess the patency of the lumen after stent implantation [7-20]. All studies use different scanning protocols and scanner types, and the reported figures for diagnostic accuracy for ISR exhibit considerable variability. Also, a multitude of stent types and stent sizes exist and have been evaluated with MDCTA, with varying results for diagnostic accuracy. It has been shown in multiple studies in vitro and in vivo that 4 detector MDCTA is not reliable for evaluation of ISR [21,22]. However 16 and 64 detector MDCTA showed a higher potential. The purpose of this study was to evaluate the pooled diagnostic accuracy of MDCTA with 16 detectors or higher for the detection of in-stent restenosis and to determine the influence of study characteristics on the diagnostic performance of MDCTA.

We tried to frame the question starting from the potential future use of MDCTA in evaluation of stent patency. A potential use that is probably reasonable is using MDCTA as a triage modality on the result of which a decision can be made whether the patient needs invasive angiography [23]. This calls for a test with a high sensitivity on a patient and stent basis, to minimize the amount of false negative patients, that are otherwise denied correct diagnosis and therapy.

## Methods

### Study selection

**Figure 1:**
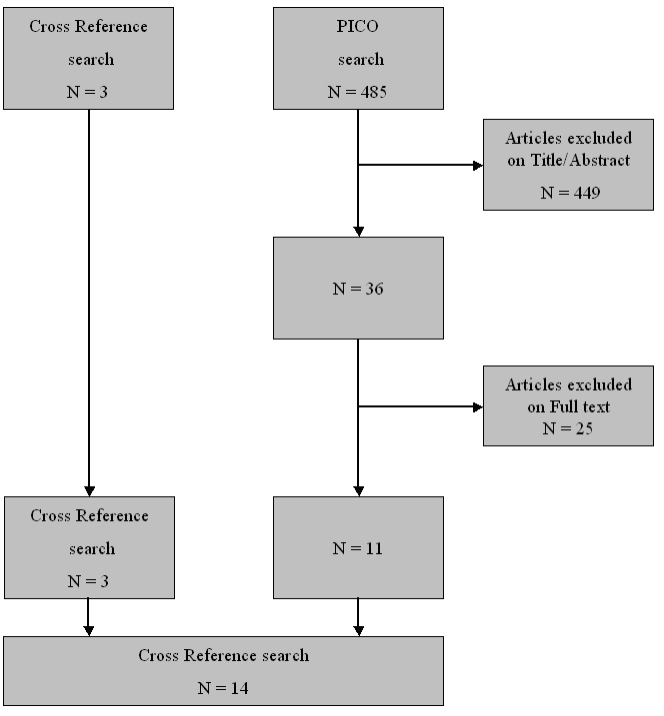
**Flow chart for the search strategy used.** Out of 485 articles found with the PICO search strategy 11 articles were included. Together with the cross reference search in total 14 articles were included.

To search for original articles, a structured search of the PUBMED database from January 1998 to March 2007 was performed using the previously described PICO search strategy [24], by three authors (PV, OB, ID). The acronym stands for: "P" = patient or group of patients, "I" = intervention, "C" = comparison intervention, and "O" = outcome. The use of the PICO strategy in the PUBMED database provides a conceptual framework for more effective searching. Entering medical subject heading (MeSH) terms in each concept of the PICO question resulted in a reference list of articles on a given topic. Although the exclusive use of MeSH terms has been described to have limitations, and other strategies or combinations have been advocated we confined ourselves to this method [25]. For this study the following MeSH terms were entered: P: coronary restenosis; I: tomography, spiral computed; C: coronary angiography; O: diagnosis. The resulting reference lists of review articles and cited articles was used to locate potential additional studies. Studies were included in the meta-analysis if they met the following inclusion criteria: patients both underwent coronary angiography (CA) and MDCTA as a follow up after stent implantation; the data were acquired with a multi-detector CT-scanner with at least 16 detectors; CA was used as the reference standard in all patients; the absolute numbers of true-positive, true-negative, false-positive and false-negative are possible to extract from the article; these absolute numbers were accepted if they were derived on a per stent basis. Exclusion criteria were: inability to obtain original numbers of false-positives (FP), false-negatives (FN), true-positives (TP), and true-negatives (TN); review article or a comment to the editor; studies not published in English; case reports; in vitro or phantom studies, and miscellaneous (Table 1). Three independent readers (PV, OB, ID) each independently evaluated the retrieved studies for possible inclusion as follows: Each investigator independently evaluated the retrieved studies for possible inclusion. In the case of conflicting findings as to whether a paper should be included, a decision was reached by consensus. In a first round articles were eliminated that clearly did not match the inclusion criteria, on the basis of the title or the abstract. In a second round, hard copies of the papers that gave rise to doubt on the basis of their abstracts were obtained and the full text was read, again eliminating a group of papers. The final group consisted of the included papers. Although quality scores have been criticized [26] we choose to guide the inclusion of studies as final gatekeeper by the quality of the study design and reporting. Formal quality assessment, Quality Assessment of Diagnostic Accuracy Studies (QUADAS, 27) was performed (third round, fig 1f). A maximum of 14 points was used to judge the quality in the final evaluation of included articles, and a score of ≥ 12 was considered acceptable.

**Table 1:** Quadas table.

	1	2	3	4	5	6	7	8	9	10	11	12	13	14
Schuijf ^7^	Y	Y	Y	Y	Y	Y	Y	Y	Y	Y	Y	Y	Y	Y
Cademartiri ^8^	Y	U	Y	Y	Y	Y	Y	Y	Y	Y	Y	Y	U	U
Gilard ^9^	Y	Y	Y	Y	Y	Y	Y	Y	Y	Y	Y	Y	U	U
Gaspar ^10^	Y	U	Y	Y	Y	Y	Y	Y	Y	Y	Y	Y	Y	U
Chabbert ^11^	Y	Y	Y	Y	Y	Y	Y	Y	Y	Y	Y	Y	Y	Y
Gilard ^12^	Y	Y	Y	Y	Y	Y	Y	Y	Y	Y	Y	Y	Y	Y
Ohnuki ^13^	Y	N	Y	Y	Y	Y	Y	Y	Y	Y	Y	Y	Y	Y
Watanabe ^14^	Y	N	Y	Y	Y	Y	Y	Y	Y	Y	Y	Y	Y	Y
Van Mieghem ^15^	Y	Y	Y	Y	Y	Y	Y	Y	Y	Y	Y	Y	Y	Y
Rist ^16^	Y	Y	Y	Y	Y	Y	Y	Y	Y	Y	Y	Y	Y	Y
Rixe ^17^	Y	Y	Y	Y	Y	Y	Y	Y	Y	Y	Y	Y	Y	Y
Kefer ^18^	Y	Y	Y	Y	Y	Y	Y	Y	Y	Y	Y	Y	Y	Y
Ehara ^19^	Y	Y	Y	Y	Y	Y	Y	Y	Y	Y	Y	Y	Y	Y
Oncel ^20^	Y	Y	Y	Y	Y	Y	Y	Y	Y	Y	Y	Y	Y	Y

For meaning of items, see [27]

### Data extraction

The study parameters were extracted first independently and subsequently by consensus if a disagreement existed between the observers concerning the numeric value of a parameter (PV, OB, ID). The absolute numbers of FN, FP, TP, TN were retrieved or calculated. The numbers were calculated with Bayes theorem if only values for sensitivity, specificity, and predictive values were reported. This was done on a per stent basis and per patient basis.

MDCTA was considered true-positive per stent if an in-stent restenosis (≥ 50% diameter stenosis) was found on MDCTA and confirmed on CA. MDCTA was considered true-negative if in-stent restenosis was correctly ruled out. MDCTA was considered false-negative if no in-stent restenosis was found on MDCTA and one was found on CA. MDCTA was considered false-positive if it revealed an in-stent restenosis and CA showed no in-stent restenosis. For the per patient analysis a patient was classified in one of these categories according to the authors decision.

### Data synthesis and statistical analysis

All values are expressed as mean value with 95% confidence intervals (95%CI) unless otherwise specified.

Interobserver agreement for study selection was evaluated with Cohen's Kappa test in which a value higher than 0.8 is considered to imply very good to excellent agreement. The different rounds of study selection were evaluated.

The analysis was performed on a per stent basis, as most studies focused on this level of information. We also did a evaluation on a per patient basis.

We evaluated potential heterogeneity and inconsistency between publications expressed with the Higgins and Thompson index [28] which calculates the I^2 ^statistic, and is a derivative of Cochran's Q [29,30]. Cochran's Q displays a low power for detection of inconsistency when the number of studies is low, and a too high power when the number of studies is high. A value of 0% indicates no observed heterogeneity, and values greater than 50% may be considered substantial heterogeneity.

Publication bias was assessed according to the method introduced by Deeks [31]. This method uses a slightly different approach, than the more classic methods [32,33] and is an optimized method suited for studies of diagnostic accuracy. A regression line for graphic analysis of publication bias is constructed. This plot is a regression of each study's effect size against some measure of its size, such as the 1/root(effective sample size), used here. The existence of publication bias can be expressed as the slope and intercept of a linear regression line between study size and effect. If the slope is not statistically different from zero, there is no publication bias.

We performed a summary receiver characteristic operating curve (SROC) analysis tailored to meta-analysis of diagnostic accuracy that takes into account any possible correlation between sensitivity and specificity in addition to within-study variation (precision) and between study variation (random effects approach). We used the bivariate meta regression approach since recent work has shown that the standard approach [34,35] can perform poorly when calculating p-values and confidence intervals [36]. Logistic meta-regression was performed with the same bivariate SROC model to evaluate the influence of several covariables on diagnostic accuracy and to identify possible causes for heterogeneity in sensitivity and specificity. The evaluated covariables included: Exclusion versus inclusion of non assessable stents expressed as a dichotomous value (yes/no), the time interval between CA and MDCT examination (number of months), brand of the scanner and the number of detectors used, the use of an edge-enhancing kernel or algorithm (soft, hard or a combination of the two), Tube voltage in Kv, strut thickness expressed as a dichotomous value (< 100 μm, ≥ 100 μm), the localization of the stent (in the left main, in all other coronary vessels) and mean diameter of stents used. The type of stent (bare metal, drug eluting) or brand of stent was not evaluated because it was impossible to homogenize the data, due to the extreme variability in the stents that were used. Tube current was not investigated either because the method of reporting varied widely. Other scan parameters were not investigated because they were reported not to have an influence in a previous meta-analysis [37]. The method of collecting and describing the data is summarized in Table 2. First, a screening for subgroups or strata defined by the covariables was done. Thereafter, some selected covariables were further investigated, depending on the presence of enough data per subgroup (at least three studies per stratum, required by our software). We focused on calculation of summary estimates of sensitivity (SE) and specificity (SP) and used likelihood ratio's for illustration of the diagnostic performance in the target group and their inherent prevalence of ISR (approx 20%). Forest plots were generated for SE and SP, and conditional likelihood graphs for likelihood ratio's and SROC curve with elliptical display of confidence intervals and prediction region. The decision not to focus on diagnostic odds ratio's was taken on the basis of the fact that we were interested in the sensitivity and specificity of the technique. Likelihood ratio's were calculated on the basis of the pooled estimates of SE and SP obtained with the bivariate SROC model [38]. The proportion of non assessable stents (NAP), defined as the ratio of non-assessable stents per number of evaluated stents was also pooled with a random effects model for meta-analysis of proportions, and a logistic meta-regression on NAP was performed with the same covariables except for one: exclusion versus inclusion of non assessable stents. All calculations were performed with STATA. (Version 10, Special Edition, StataCorp, 4905 Lakeway Drive, College Station, Texas 77845 USA.)

**Table 2:** Covariables for logistic (meta-)regression.

Author	Ndet	brand	Kernel	CT/PCI	NAP	StrTh	EXC	location	size	Age	Kv
Schuijf 7	1	4	0	14	0.23	1	0	2	-	62	120
Cademartiri 8	1	1	0	6	0.02	0	1	2	-	60	120
Gilard 9	1	3	0	6	0.07	0	1	1	3.9	63	120
Gaspar 10	2	3	0	0	0.05	2	0	2	3.3	63	120
Chabbert 11	1	1	4	6	0.26	0	0	2	3.25	67.4	120
Gilard 12	1	3	0	12	0.46	0	1	2	3.13	68	120
Ohnuki 13	1	1	2	0	0.00	0	0	2	3.32	65	120
Watanabe 14	1	1	1	6	0.17	0	1	2	3.3	64	120
Van Mieghem 15	3	1	2	8	0.09	0	1	1	3	61	120
Rist 16	4	1	4	1	0.02	2	1	2	3.16	59	120
Rixe 17	4	1	2	13	0.42	2	0	2	2.97	58	120
Kefer 18	1	3	2	12	0.07	0	1	2	2.8	64	140
Ehara 19	4	1	2	3	0.00	1	0	2	3.27	67	120
Oncel 20	4	1	2	20.1	0.00	0	0	2	3.17	58.2	120

Ndet: number of detectors. 1 = 16, 2 = 40, 3 = 16/64, 4 = 64

Brand: 1 = Siemens, 2 = GE, 3 = Philips, 4 = Toshiba

Kernel: 1 = B20-30f, 2 = B46f, 3 = B46, f + filter, 4 = combination

CT/PCI: average number of months between scan and stent placement

NAP: Non-assessable proportion of stents

StrTh: Strut Thickness. 0 = no data, 1 = Str Th < 100 μm, 2 = Str Th > 100 μm

EXC: non-assessable stents were excluded before analysis: 1, not excluded: 0

Loc: Stent location in the coronary. Left main or not specified = 1, other = 2

Size: average stent diameter in mm

Kv: kilovoltage

## Results

### Study selection and data extraction

The PUBMED search for original articles resulted in 485 articles. 449 articles were excluded on the basis of their title or abstract with 36 remaining for further evaluation. From these 36 articles, 11 were finally included in the meta-analysis [7,10-12,14-20]. Three additional articles found on the basis of cross references were also finally included in the meta-analysis [8,9,13]. No studies were excluded on the basis of QUADAS when considering this final group. QUADAS was scored with yes, no or unclear for the criterium investigated and a table was constructed (Table 1).

The final group of studies consisted of 4 studies on 64-detector CT, 1 on 40-detector CT, 1 on a combination of 64- and 16-detector CT, and 8 on 16-detector CT. 24 studies were excluded. Number and reasons for exclusion are found in Table 1. A Flow diagram of the review process is given in figure 1. In additional file 1 the studies that were excluded were cited and classified according to Table 3.

Interobserver agreement (Cohen's Kappa) for the selection of articles between the three readers was respectively 0.75, 0.87 and 1.0 for the different search rounds.

**Table 3:** Numbers and reasons for exclusion

Reason for exclusion	
Case Report	9
Comment to the editor	1
No English	3
Review Article	2
In Vitro/Phantom studies	2
Unability to obtain FN, FP, TN, TP	6
Miscellaneous	2
Total	25

FN: False Negative

FP: False Positive

TN: True Negative

TP: True Positive

Important study characteristics are displayed in Table 2.

### Data synthesis and statistical analysis

A total of 1039 stents were analyzed. In the per patient analysis 400 patients were analysed.

Raw data from the included studies are displayed in Table 4 (per stent analysis) an 5 (per patient analysis).

**Table 4:** Raw data on a stent basis.

Author	Journal	Year	FP	TP	TN	FN	SE (95%CI)		SP (95%CI)	
Schuijf 7	Am J Card	2004	15	7	41	2	0.78	0.40	0.73	0.60
							0.97		0.84	
Cademartiri 8	Am J Card	2005	1	5	67	1	0.83	0.36	0.99	0.92
							1.00		1.00	
Gilard 9	Am J Card	2005	2	2	25	0	1.00	0.16	0.93	0.76
							1.00		0.99	
Gaspar 10	JACC	2005	11	14	78	8	0.64	0.41	0.88	0.79
							0.83		0.94	
Chabbert 11	Eur Rad	2006	28	21	57	2	0.91	0.72	0.67	0.56
							0.99		0.77	
Gilard 12	Heart	2006	0	10	108	4	0.71	0.42	1.00	0.97
							0.92		1.00	
Ohnuki 13	Int J card	2006	2	3	14	1	0.75	0.19	0.88	0.62
							0.99		0.98	
Watanabe 14	circulation	2006	2	6	27	0	1.00	0.54	0.93	0.77
							1.00		0.99	
Van Mieghem 15	circulation	2006	5	10	55	0	1.00	0.69	0.92	0.82
							1.00		0.97	
Rist 16	Acad Radiol	2006	3	6	34	2	0.75	0.35	0.92	0.78
							0.97		0.98	
Rixe 17	Eur Heart Jnl	2006	39	6	51	6	0.50	0.21	0.57	0.46
							0.79		0.67	
Kefer 18	Eur Rad	2007	1	12	50	6	0.67	0.41	0.98	0.90
							0.87		1.00	
Ehara 19	JACC	2007	19	22	82	2	0.92	0.73	0.81	0.72
							0.99		0.88	
Oncel 20	Radiology	2007	1	17	19	2	0.89	0.67	0.95	0.75
							0.99		1.00	

FP: False positive

TP: True positive

TN: True negative

FN: False negative

CI: Confidence intervals

**Table 5:** Raw data on a patient basis.

Author	FP	TP	TN	FN	SE (95%CI)		SP(95%CI)	
Schuijf 7	15	7	41	2	0.77	0.400 – 0.97	0.73	0.59 – 0.84
Gilard 9	2	2	25	0	1.00	0.15 – 1.00	0.92	0.75 – 0.99
Gaspar 10	11	14	78	8	0.63	0.40 – 0.82	0.87	0.79 – 0.93
Van Mieghem 15	5	10	55	0	1.00	0.69 – 1.00	0.91	0.81 – 0.97
Ehara 19	19	22	82	2	0.91	0.73 – 0.99	0.81	0.72 – 0.88

FP: False positive

TP: True positive

TN: True negative

FN: False negative

CI: Confidence intervals

Substantial inconsistency between studies was found when calculating the pooled SE and SP on a stent basis with the bivariate model (I^2^, 91.42%, 79.51%, respectively).

There was no substantial publication bias: Per stent: Slope 4.62 (p, 0.67), Intercept 3.10 (p, 0.09 Per patient: slope 3.62 (p, 0.81), Intercept 2.96 (p, 0.24)

**Figure 2:**
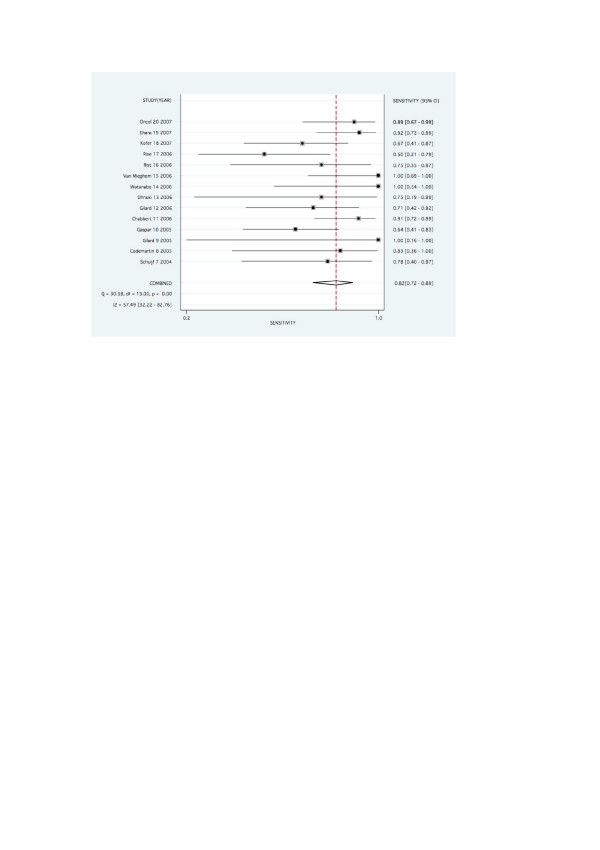
Forest plot for pooled sensitivity per stent.

**Figure 3:**
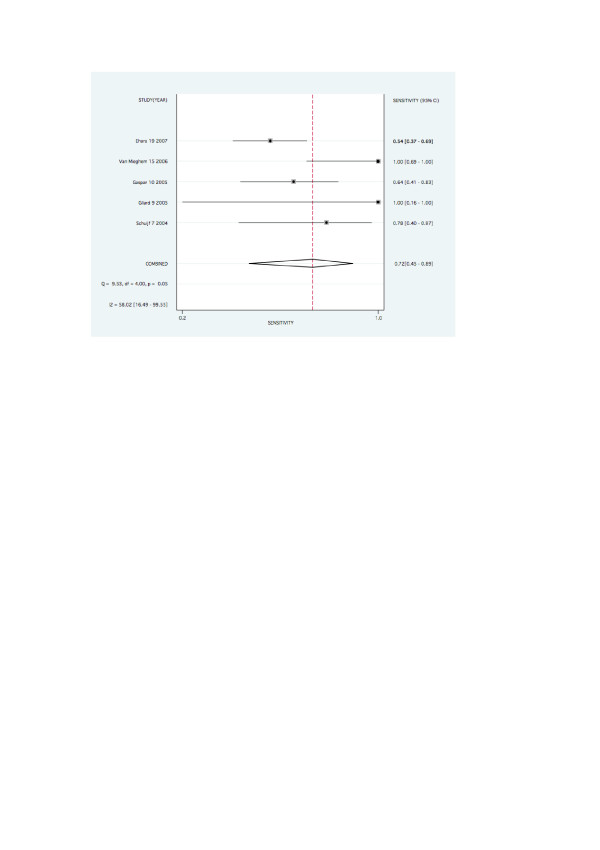
Forest plot for pooled sensitivity per patient.

The pooled values for detecting significant ISR on a stent basis were as follows: Pooled sensitivity and specificity were 0.82 (0.72–0.89) and 0.91 (0.83–0.96). Pooled negative likelihood ratio and positive likelihood ratio were 0.20 (0.13–0.32) and 9.34 (4.68–18.62) respectively. Values on a patient basis are found in figures 2 and 3.

**Figure 4:**
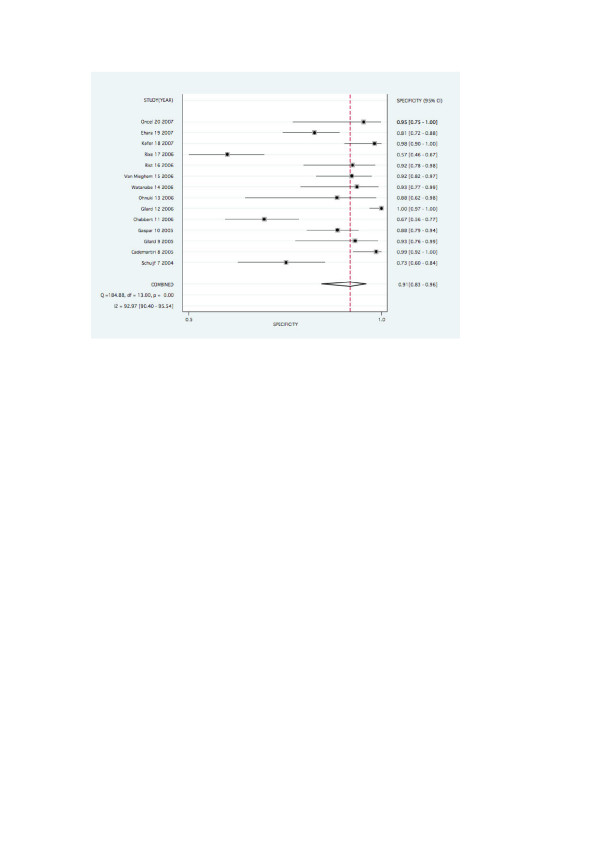
Forest plot for pooled specificity per stent.

**Figure 5:**
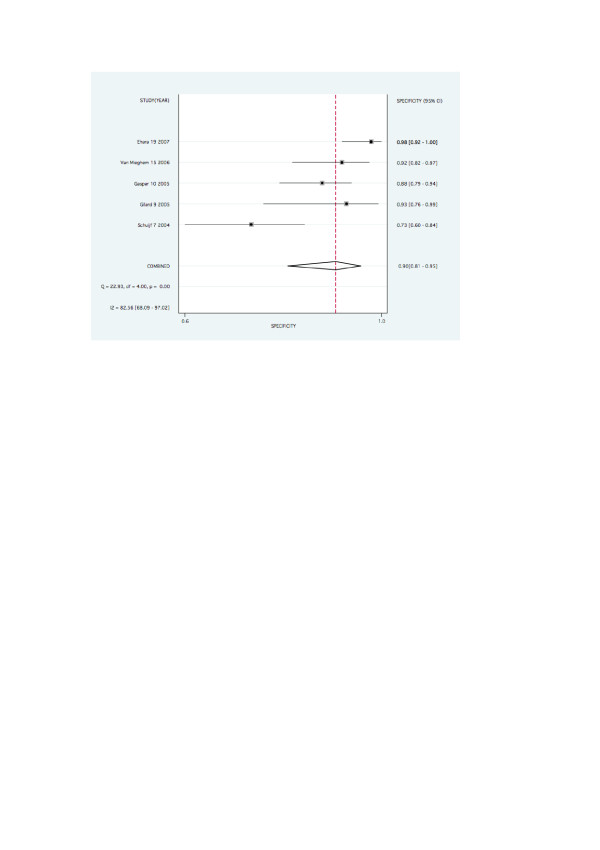
Forest plot for pooled specificity per patient.

Forest plots for pooled sensitivity, specificity, are graphed in figures 2, 3, 4 and 5.

**Figure 6:**
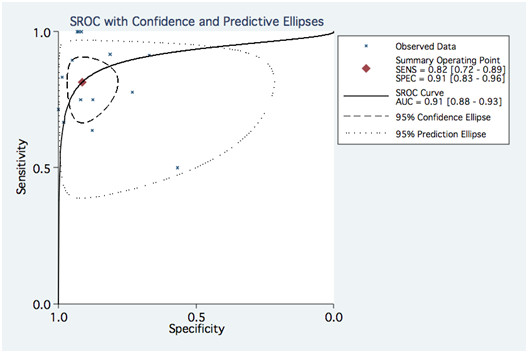
**SROC from the bivariate model for pooled data per stent**. SENS: sensitivity, SPEC: Specificity, AUC: Area under the curve.

**Figure 7:**
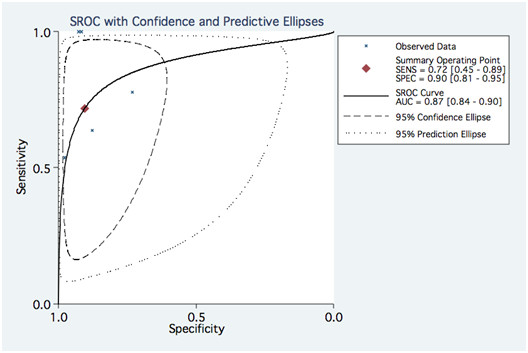
**SROC from the bivariate model for pooled data per patient**. SENS: sensitivity, SPEC: Specificity, AUC: Area under the curve.

**Figure 8:**
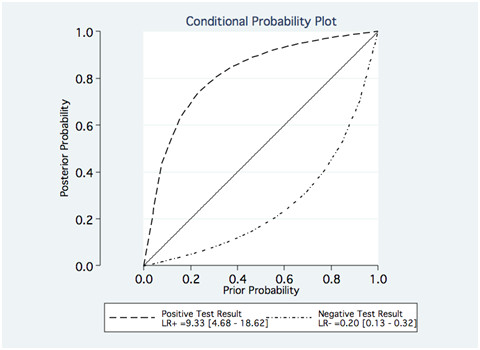
**Conditional Probability plots per stent**. LR+: positive likelihood ratio, LR-: negative likelihood ratio.

**Figure 9:**
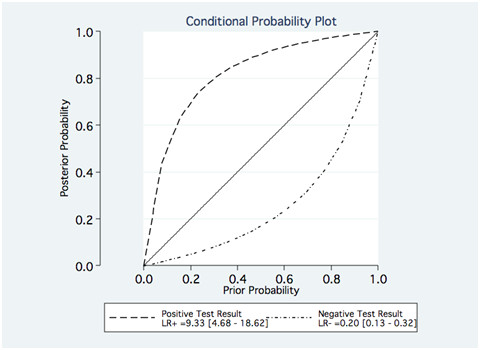
**Conditional Probability plots per patient**. LR+: positive likelihood ratio, LR-: negative likelihood ratio.

Random effects SROC curve and conditional probability plots are displayed in figure 6, 7, 8 and 9. The results of meta-regression are outlined in Table 6. Using a threshold of p < 0.05 for statistical significance, none of the evaluated covariables were significant predictors of sensitivity. The only significant predictor of specificity was the exclusion of non-assessable segments (p 0.003). A few selected covariables that were amenable to meaningful subgroup analysis are displayed with their stratum specific sensitivities and specificities in Table 7. Logistic metaregression on a per patient basis showed no predicting covariables. The pooled NAP was 0.11 (95%CI, 0,04–0,20, range 0.00–0.46). Meta-regression on NAP showed that it was influenced by the number of detectors, stent diameter, strut thickness and the use of an edge-enhancing kernel (Table 8).

**Table 6:** Results for the bivariate logistic meta-regression on diagnostic performance: per stent analysis and detection of subgroups.

Covariable	Sensitivity (95%CI)	p value	Specificity (95%CI)	p value
Year of publication	0.84 [0.71–0.92]	0.64	0.93 [0.81–0.98]	0.72
No of detectors	0.82 [0.70–0.89]	0.97	0.88 [0.76–0.95]	0.57
Brand	0.74 [0.62–0.84]	0.29	0.93 [0.83–0.97]	0.74
Kernel	0.84 [0.73–0.90]	0.65	0.88 [0.76–0.94]	0.52
CTPCI	0.80 [0.69–0.88]	0.91	0.91 [0.82–0.96]	0.99
Strth	0.78 [0.69–0.85]	0.14	0.89 [0.79–0.94]	0.16
Exc	0.83 [0.67–0.93]	0.72	0.97 [0.93–0.98]	0.00
Location	0.00 [0.00–1.00]	1.00	0.89 [0.46–0.99]	0.82
Size	0.98 [0.62–1.00]	0.19	0.88 [0.24–0.99]	0.86
Age	0.79 [0.68–0.87]	0.84	0.93 [0.86–0.97]	0.74
Kv	0.81 [0.72–0.88]	0.91	0.92 [0.85–0.96]	0.85

If p-value is below 0.05 significant subgroups exist attributable to the investigated covariable. Sensitivity and specificity is given for the most deviating stratum with p-value comparing the values with the complete group without stratification. Only one covariable results in significant difference between strata: specificity is significantly different when grouping studies that excluded or included non-assessable segments. See also table 6 that compares some stratum specific sensitivities and specificities.

Legend for covariables see table 3

95% CI: 95% Confidence intervals

**Table 7:** Results of bivariate analysis with covariables, per stent analysis.

Study characteristic	No of studies	sensitivity	95% CI	specificity	95% CI
All studies	14	0.82	0.72–0.89	0.91	0.83–0.96
Number of detectors					
64	4	0.83	0.71–0.90	0.89	0.70–0.97
lower	10	0.81	0.68–0.89	0.93	0.85–0.97
Non assessable excluded					
yes	7	0.84	0.66–0.93	0.97	0.93–0.99
no	7	0.81	0.66–0.90	0.79	0.68–0.87
Year of publication					
2004 or earlier	4	0.73	0.55–0.85	0.91	0.77–0.97
		
later than 2004	10	0.84	0.72–0.91	0.91	0.80–0.97
Time between MDCTA and PCI					
less than 6 months	8	0.83	0.69–0.92	0.89	0.81–0.92
6 months or longer	5	0.72	0.56–0.84	0.93	0.67–0.99
Brand					
Siemens	9	0.69	0.57–0.79	0.95	0.80–0.99
Philips or Toshiba	5	0.88	0.76–0.94	0.88	0.77–0.94

Stratum specific sensitivities and specificities were compared for some characteristics that were amenable to meaningful analysis of subgroups (arbitrary value of more than 3 studies per subgroup)

**Table 8:** Results of logistic meta regression on NAP.

	**Coeff**	**p-value**	**rDOR**	**95%CI**
**Intercept**	10.49	0.00		
**N detectors**	-1.39	0.00	0.25	0.16–0.39
**Stent diameter**	-2.06	0.00	0.13	0.04–0.37
**Strut Thickness**	0.71	0.00	2.03	1.43–2.89
**Kernel**	-0.46	0.00	0.63	0.51–0.79

Coeff: Coefficient

rDOR: Relative diagnostic odds ratio

95% CI: 95% Confidence intervals referring to RDOR.

## Discussion

The results of this study showed that pooled sensitivity and specificity on a stent basis were 0.82 (0.72–0.89) and 0.91 (0.83–0.96) which is moderate (sensitivity) to very good (specificity). Considerable inconsistency/heterogeneity was found between all included studies. There was no difference in the diagnostic performance between scanners with 16 or 64 detectors and the only variable that showed to have influence on diagnostic performance was the exclusion of non-assessable stents before analysis. The proportion of stents that was non assessable showed a very important variability, and was in one study as high as 0.46 and showed a pooled value of 0.11. Meta-regression on NAP showed that it was influenced by the number of detectors, stent diameter, strut thickness and the use of an edge-enhancing kernel. This is probably reflecting evolving stent technology, as will be stated later in this discussion

Pooled negative likelihood ratio and positive likelihood ratio were 0.20 (0.13–0.32) and 9.34 (4.68–18.62) respectively. For a test to be helpful in diagnosis, it is generally accepted that LR+ should be higher than 10 and LR- below 0.1 [39].

These results underscore the fact that demonstration of ISR with MDCTA can be done but still suffers from some problems, and that non-invasive imaging of stents to rule out ISR (moderate sensitivity) remains a challenge, even after the addition of 16 to 64 detector MDCTA to the diagnostic armentarium. The sensitivity is probably not good enough to use the technique as a rule-out triage modality.

On the basis of studies in vitro, a logical and intuitively attractive concept has been formulated on stent imaging with MDCTA [21,22]. Thick struts, stent size and stent material were found to have an influence on the capability of MDCTA to visualize the stent lumen. Smaller stents, thicker struts and dense stent material (bare metal) were more difficult to image without beam-hardening artefacts and blooming. This concept (except for stent material) could be confirmed by the pooled analysis in this study. The influence was not directly on diagnostic performance but through the number of non assessable segments, that was higher in stents with a smaller diameter and thicker struts. One possible explanation that the possible effect of stent material could not be confirmed is that it was difficult to identify groups of identical stents which resulted in a statistical lack of power.

In addition to these stent related factors, scan acquisition parameters (number of detectors and the use of an edge enhancing kernel) had an influence, also through the number of non-assessable segments.

Although these principles suggest that stent imaging with MDCTA is not always straightforward and prone to failure in certain stents, the general trend in interventional cardiology to use stents with thinner struts and less dense material will probably obviate at least some of this problem. Also scanner technology is advancing quickly and some of the scanner related problems will probably also be obviated in the future.

As said previously, non-invasive evaluation of coronary artery disease after stent placement is a clinical challenge, and CA is not recommended as a routine follow-up technique due to the associated risks and cost. Therefore, non-invasive follow-up of ISR and re-occurrence of myocardial ischemia has been investigated with a variety of techniques. ECG stress testing has been tested and in a recent overview, sensitivity and specificity was 54% and 70% respectively, which is clearly insufficient for a triage modality [40]. Myocardial perfusion imaging with nuclear techniques and with contrast echocardiography has been documented previously [41,42]. Sensitivity and Specificity for the diagnosis of regional restenosis was 73% and 75% respectively in the contrast echo study and 76% and 83% in the 99 m technetium tetrofosmin myocardial perfusion imaging, values which are inferior to MDCTA on a per stent basis and almost equal on per patient basis, but both worse for specificity on a stent an patient basis than the pooled diagnostic performance with MDCTA. MRI has also been tested for this purpose, but imaging of stents remains only possible in certain stents, no formal data on diagnostic performance have been published and experience remains largely anecdotical [43-45].

We have to acknowledge some limitations in this study.

The rather small size of the individual cohorts and the methodology (examining very different stents and stent sizes in each study) resulted in a heterogenous group of stents that make it cumbersome to reliably pool the data. This makes it also harder to obtain reliable results with the technique of multivariable logistic meta-regression, due to the very high number of explanatory variables (different stents) involved.

Some clear disadvantages of MDCTA are the relatively high radiation dose that goes with the examination, with average doses ranging from 10 to 20 mS [46], and the influence of an irregular rhythm [47,48] on the diagnostic performance of MDCTA.

Since MDCTA technology and stent technology is advancing very rapidly, future studies should focus on the use of state-of the art equipment both in terms of scan methodology and in terms of the stents investigated. If ISR is studied in patients, rigorous methodology to group enough stents of the same size and type should be done, preferably in larger and important anatomical locations. This would probably be very helpful to predict which patients and stents are good candidates to be investigated with MDCTA, with a reasonable change of obtaining images that can be reliably interpreted.

Pooled analysis of diagnostic performance of MDCTA of ISR shows that the technique is probably useful, but that more thorough and uniform investigation of modern stents with the latest equipment will probably be needed to shed more light on the clinical usefulness of this technique. The diagnostic performance is influenced by the method of reporting (exclusion of non-assessable stents). The proportion of non-assessable stents is influenced by the number of detectors, stent diameter, strut thickness and the use of an edge-enhancing kernel.

## Conclusion

The sensitivity of MDTCA for the detection of in-stent stenosis is insufficient to use this test to select patients for further invasive testing as with this strategy around 20% of the patients with in-stent stenosis would be missed. Further improvement of scanner technology is needed before it can be recommended as a triage instrument in practice. In addition, the number of non-assessable stents is also high.

## Competing interests

The authors declare that they have no competing interests.

## Authors' contributions

PKV carried out the literature search, statistical analysis and drafted the manuscript. ID carried out the literature search, statistical analysis and drafted the manuscript. OB, GS and EV participated in the study design and collection of data. WW conceived of the study and participated in its design and coordination. BAD participated in the statistical analysis and coordinated methodologic aspects. All authors read and approved the final manuscript.

## Pre-publication history

The pre-publication history for this paper can be accessed here:



## Supplementary Material

Additional file 1 Appendix 1 studies that were excluded according to reason for exclusion. Click here for file
