# Supplementary material for: Multidetector computed tomography angiography for assessment of in-stent restenosis: meta-analysis of diagnostic performance
Source: BMC Med Imaging. 2008 Jul 31;8:14. doi: 10.1186/1471-2342-8-14 (PMC2533305; doi:10.1186/1471-2342-8-14)
Supplement: Additional file 1 — Appendix 1 studies that were excluded according to reason for exclusion. [file 1471-2342-8-14-S1.doc]

**Appendix 1. Articles excluded from the study**

### Case report (n=9)

N. Funabashi, N. Komiyama, and I. Komuro. Patency of the right coronary artery following implantation of metallic stent demonstrated by multislice computed tomography. Heart 2005; 91 :36.

N. Funabashi, F. Maeda, K. Nakamura, et al. Patency of the left coronary artery by 64-slice multislice computed tomography following implantation of sirolimus-eluting stent. Int.J Cardiol 2006; 111:333-335.

M. H. Jim, K. H. Yiu, and W. H. Chow. In-stent restenosis in idiopathic isolated ostial left main coronary artery stenosis. Int.J Cardiol 2007; 114 :e111-e113.

J. Y. Kim, J. Yoon, H. S. Jung, B. S. Yoo, and S. H. Lee. Percutaneous coronary stenting in guide-induced aortocoronary dissection: angiographic and CT findings. Int.J Cardiovasc Imaging 2005; 21:375-378.

M. R. Mohaved. Six-months patency of three long drug eluting stents documented by surveillance coronary multi-detector computed tomography (MDCT). Clin.Res.Cardiol 2006; 95:605-609.

S. Notaristefano, C. Giombolini, S. Santucci, F. Fortunati, K. Savino, A. Notaristefano, and G. Ambrosio. Successful treatment by percutaneous stent deployment of severe retrograde dissection of the right coronary artery extending into the sinus of Valsalva and ascending aorta. Int.J Cardiol 2005;104:112-114.

M. Otsuka, S. Hirohashi, M. Watanabe, S. Uemura, and K. Kichikawa. [Follow-up coronary angiography with multi-slice computed tomography after stenting]. J Cardiol 2002; 40:283-286.

D. M. Sado, R. K. Bull, and J. R. Radvan. Computed tomography to visualise a left coronary artery main stem stent. Heart 2006; 92:1759.

J. D. Schuijf, J. J. Bax, J. W. Jukema, H. J. Lamb, M. S. Dirksen, E. E. van der Wall, and Roos A. de. Coronary stent imaging with multidetector row computed tomography. Int.J Cardiovasc.Imaging 2004; 20:341-344.

### Comment to the editor (n=1)

C. Kimmelstiel. Multislice computed tomography after left main drug-eluting stenting: are we putting the cart before the horse? Circulation 2006; 114:616-619.

### No English article (n=3)

K. Anami, H. Anno, A. Hayashiguchi, et al. [Visualization of coronary artery stents by MSCT at 0.5-mm slice thickness]. Nippon Hoshasen Gijutsu Gakkai Zasshi 2004; 60:278-285.

L. Y. Kong, Z. Y. Jin, Y. N. Wang, et al. [Assessment of coronary stent lumen visibility and patency by 64-slice spiral CT angiography]. Zhongguo Yi.Xue.Ke.Xue.Yuan Xue.Bao 2006; 28 :32-35.

M. Pasowicz, P. Klimeczek, T. Przewlocki, et al. [Evaluation of patency of coronary artery bypass grafts and stents using multislice spiral computed tomography in comparison with angiography]. Przegl.Lek 2002; 59:616-619.

### Review (n=2)

R. Haas. Implantation and imaging of coronary stents. Radiol.Technol 1996; 67 :233-244.

K. H. Soon, A. M. Kelly, N. Cox, I. Chaitowitz, K. W. Bell, and Y. L. Lim. Non-invasive multislice computed tomography coronary angiography for imaging coronary arteries, stents and bypass grafts. Intern.Med.J 2006; 36:43-50.

### In Vitro/ phantom (n= 2)

K. Nieman, F. Cademartiri, R. Raaijmakers, P. Pattynama, and Feyter P. de. Noninvasive angiographic evaluation of coronary stents with multi-slice spiral computed tomography. Herz 2003; 28:136-142.

D. Maintz, H. Seifarth, T. Flohr, et al. Improved coronary artery stent visualization and in-stent stenosis detection using 16-slice computed-tomography and dedicated image reconstruction technique. Invest Radiol 2003; 38:790-795.

### No diagnostic accuracy (n= 6)

C. Hong, G. S. Chrysant, P. K. Woodard, and K. T. Bae. Coronary artery stent patency assessed with in-stent contrast enhancement measured at multi-detector row CT angiography: initial experience. Radiology 2004; 233:286-291.

T. Kitagawa, T. Fujii, Y. Tomohiro, et al. Noninvasive assessment of coronary stents in patients by 16-slice computed tomography. Int.J Cardiol 2006; 109:188-194.

G. Ligabue, R. Rossi, C. Ratti, M. Favali, M. G. Modena, and R. Romagnoli. Noninvasive evaluation of coronary artery stents patency after PTCA: role of Multislice Computed Tomography. Radiol.Med.(Torino) 2004; 108:128-137.

D. Maintz, M. Grude, E. M. Fallenberg, W. Heindel, and R. Fischbach. Assessment of coronary arterial stents by multislice-CT angiography. Acta Radiol 2003; 44:597-603.

M. Pasowicz, P. Klimeczek, P. Pieniazek, et al. Assessment of stent patency using multi-slice spiral computed tomography: initial experience. Acta Cardiol 2002; 57:63-64.

T Sheth, J Dodd, U Hoffmann, et al. Coronary stent assessability by 64 slice multi-detector computed tomography: Cath Card Int 2007; 69:933-938.

### Miscellaneous (n= 1)

S. Kruger, A. H. Mahnken, A. M. Sinha, et al. Multislice spiral computed tomography for the detection of coronary stent restenosis and patency. Int.J Cardiol 2003; 89:167-172.

H. Seifarth, R. Raupach, S. Schaller, et al. Assessment of coronary artery stents using 16-slice MDCT angiography: evaluation of a dedicated reconstruction kernel and a noise reduction filter. Eur.Radiol 2005; 15:721-726.
